# Four Eating Behaviors That Might Prevent Metabolic Syndrome Onset in Older Adults: Cohort Study

**DOI:** 10.1093/geroni/igaa057.776

**Published:** 2020-12-16

**Authors:** Sayori Wada, Masahide Hamaguchi, Nami Nakabe, Miho Ueda

**Affiliations:** 1 Kyoto Prefectural University, Kyoto, Japan; 2 Kyoto Prefectural University of Medicine, Kyoto, Japan; 3 Japanese Red Cross Kyoto Daiichi Hospital, Kyoto, Japan

## Abstract

Metabolic syndrome (MetS) is associated with cardiovascular disease and cancer, the leading causes of mortality and morbidity. Although eating behaviors may have an impact on the risk of MetS, exactly which behaviors can prevent MetS is not fully elucidated. We evaluated the onset of MetS in relation to eating behaviors among Japanese older adults aged 65 to 93. We enrolled individuals who underwent health check-ups between April 2008 and March 2019, and performed a nine-year follow-up in this cohort study. Cox regression models were used to compare hazard ratios for MetS onset. Among the 2,661 older adults included, the mean age was 70.21 ± 0.089 years and 46% were women. During a mean follow-up of 1567.3 ± 19.3 months, 499 candidates (18%) developed MetS. The risk of MetS was significantly low in subjects in the “often eat vegetables” and “eat more than 30 items daily” groups (hazard ratio (HR) (95% confidence interval (CI)): 0.721 (0.595–0.872), p = 0.001; and 0.690 (0.545–0.874), p = 0.002, vs without the behavior, respectively). On the contrary, the risk of MetS was significantly higher in subjects in the “eat quickly” and “eat out more than twice a day” groups (HR (95% CI): 1.442 (1.208–1.721), p < 0.001; and 1.534 (1.245–1.890), p < 0.001, vs without the behavior, respectively). Four eating behaviors—regular vegetable consumption, eating more than 30 items daily, eating slowly, and refraining from eating out too often—might be beneficial with regard to preventing the onset of MetS.

